# Epigenetic Modulation and Bone Metastasis: Evolving Therapeutic Strategies

**DOI:** 10.3390/ph18081140

**Published:** 2025-07-31

**Authors:** Mahmoud Zhra, Jasmine Hanafy Holail, Khalid S. Mohammad

**Affiliations:** 1 Department of Anatomy and Genetics, College of Medicine, Alfaisal University, Riyadh 11533, Saudi Arabia; mzahra@alfaisal.edu; 2 Department of Biochemistry and Molecular Medicine, College of Medicine, Alfaisal University, Riyadh 11533, Saudi Arabia; jholail@alfaisal.edu

**Keywords:** bone metastasis, epigenetic therapy, DNA methylation, histone modification, non-coding RNAs, tumor microenvironment, bone-targeting agents

## Abstract

Bone metastasis remains a significant cause of morbidity and diminished quality of life in patients with advanced breast, prostate, and lung cancers. Emerging research highlights the pivotal role of reversible epigenetic alterations, including DNA methylation, histone modifications, chromatin remodeling complex dysregulation, and non-coding RNA networks, in orchestrating each phase of skeletal colonization. Site-specific promoter hypermethylation of tumor suppressor genes such as *HIN-1* and *RASSF1A*, alongside global DNA hypomethylation that activates metastasis-associated genes, contributes to cancer cell plasticity and facilitates epithelial-to-mesenchymal transition (EMT). Key histone modifiers, including *KLF5*, *EZH2*, and the demethylases KDM4/6, regulate osteoclastogenic signaling pathways and the transition between metastatic dormancy and reactivation. Simultaneously, SWI/SNF chromatin remodelers such as BRG1 and BRM reconfigure enhancer–promoter interactions that promote bone tropism. Non-coding RNAs, including miRNAs, lncRNAs, and circRNAs (e.g., miR-34a, NORAD, circIKBKB), circulate via exosomes to modulate the RANKL/OPG axis, thereby conditioning the bone microenvironment and fostering the formation of a pre-metastatic niche. These mechanistic insights have accelerated the development of epigenetic therapies. DNA methyltransferase inhibitors (e.g., decitabine, guadecitabine) have shown promise in attenuating osteoclast differentiation, while histone deacetylase inhibitors display context-dependent effects on tumor progression and bone remodeling. Inhibitors targeting EZH2, BET proteins, and KDM1A are now advancing through early-phase clinical trials, often in combination with bisphosphonates or immune checkpoint inhibitors. Moreover, novel approaches such as CRISPR/dCas9-based epigenome editing and RNA-targeted therapies offer locus-specific reprogramming potential. Together, these advances position epigenetic modulation as a promising axis in precision oncology aimed at interrupting the pathological crosstalk between tumor cells and the bone microenvironment. This review synthesizes current mechanistic understanding, evaluates the therapeutic landscape, and outlines the translational challenges ahead in leveraging epigenetic science to prevent and treat bone metastases.

## 1. Introduction

Bone metastasis is a prevalent and serious complication of advanced cancers, notably breast, prostate, and lung cancers. It occurs when cancer cells disseminate from their primary sites to the bone, leading to skeletal-related events (SREs) such as pain and fractures, which significantly increase morbidity and adversely affect patient survival and quality of life [1]. The bone microenvironment plays a crucial role in the metastatic process, providing a unique niche that supports the survival, proliferation, and dissemination of tumor cells. This environment comprises various cellular components, including osteoblasts and osteoclasts, whose interactions with tumor cells disrupt normal bone remodeling, leading to osteolytic or osteoblastic lesions [2].

Osteoclasts facilitate tumor invasion and metastasis by breaking down bone tissue, while osteoblasts build new bone and potentially support metastatic tumor growth [3]. In osteolytic metastases, commonly seen in breast cancer, increased osteoclast activity leads to bone resorption, releasing growth factors that further promote tumor growth. Conversely, osteoblastic metastases, often associated with prostate cancer, involve heightened osteoblast activity, resulting in abnormal bone formation [4]. A critical pathway in this process is the OPG-RANKL-RANK axis, which regulates osteoclast differentiation and activity [5]. Tumor cells can manipulate this pathway to enhance bone resorption, creating a “vicious cycle” where bone degradation releases growth factors and calcium that support further tumor proliferation [3]. Additionally, signaling pathways such as TGF-β and Wnt play a pivotal role in regulating tumor cell dormancy and reactivation within the bone marrow microenvironment. These pathways determine whether disseminated tumor cells remain dormant or resume proliferation, impacting disease progression and treatment outcomes [6].

Recent research highlights the critical role of epigenetic modifications in cancer progression and metastasis. These heritable and reversible changes in gene expression occur without alterations to the DNA sequence and encompass mechanisms such as DNA methylation, histone modifications, chromatin remodeling, and non-coding RNA regulation [7,8]. DNA methylation can silence tumor suppressor genes, facilitating uncontrolled cell proliferation [9]. Histone modifications alter chromatin structure, influencing gene accessibility and thereby impacting gene expression patterns [10]. Chromatin remodeling dynamically restructures chromatin architecture, affecting the transcriptional landscape [11]. Non-coding RNAs, including microRNAs and long non-coding RNAs, modulate gene expression at various levels, contributing to tumor development and metastasis [12].

This intricate network of epigenetic regulation influences not only tumor cells but also the surrounding microenvironment, fostering conditions conducive to metastasis. These modifications significantly alter gene expression patterns, enhancing cellular behaviors that enable cancer cells to invade adjacent tissues and establish metastatic niches [13,14]. Additionally, these epigenetic alterations contribute to epithelial–mesenchymal transition (EMT), a key process in cancer metastasis [10,15]. EMT involves the downregulation of epithelial markers and upregulation of mesenchymal markers, enhancing the migratory and invasive capabilities of cancer cells [16]. EMT-inducing transcription factors often collaborate with chromatin-modifying proteins to orchestrate these gene expression changes [17].

The dynamic and reversible nature of epigenetic modifications presents promising avenues for therapeutic intervention. Epigenetic drugs, such as DNA demethylating agents and histone deacetylase inhibitors, have shown potential in reversing aberrant epigenetic states, thereby inhibiting cancer progression and metastasis [18]. However, challenges remain in translating these therapies into clinical settings. The complexity of tumor heterogeneity and the dynamic nature of epigenetic modifications complicate treatment approaches, necessitating a comprehensive understanding of individual tumor epigenomes [19]. Importantly, epigenetic modifications represent a vital layer within multi-omics approaches, providing reversible regulatory insights that complement genomic and transcriptomic data. Integrating epigenomic information enhances the understanding of metastatic mechanisms and improves the identification of novel biomarkers and therapeutic targets in bone metastasis [20,21].

Given the critical role of epigenetic modifications in bone metastasis, there is a strong case for targeting these pathways in treatment strategies. Epigenetic therapies offer a promising approach to reprogram cancer cells and potentially reverse abnormal gene expression patterns linked to metastasis. By identifying specific epigenetic changes associated with bone metastasis, we can develop targeted therapies that may improve patient outcomes and enhance diagnosis and prognosis.

In this review, we will explore the relationship between epigenetic modifications and bone metastasis. We will discuss different types of epigenetic changes, their effects on tumor biology, and their role in the bone microenvironment. We will also examine current therapeutic approaches, challenges in clinical applications, and future research directions. Our goal is to provide insights that can inform future studies and enhance clinical practices for managing bone metastases.

To ensure a comprehensive and insightful overview of the latest evidence, we conducted a structured literature search using PubMed and Google Scholar. The search employed combinations of keywords such as “bone metastasis”, “epigenetic modifications”, epigenetic therapy”, “DNA methylation”, “histone modification”, “non-coding RNAs”, “chromatin remodeling”, and “bone microenvironment.” We included English-language, peer-reviewed studies offering mechanistic insights, preclinical or clinical evaluations, or therapeutic implications relevant to bone metastases. Both foundational and recent studies were considered to provide historical context and highlight current trends in epigenetic modulation of bone metastasis. Applicable clinical trials were identified via ClinicalTrials.gov. Studies lacking sufficient mechanistic or clinical detail were excluded.

## 2. Epigenetic Landscape of Bone Metastasis

**Figure 1:**
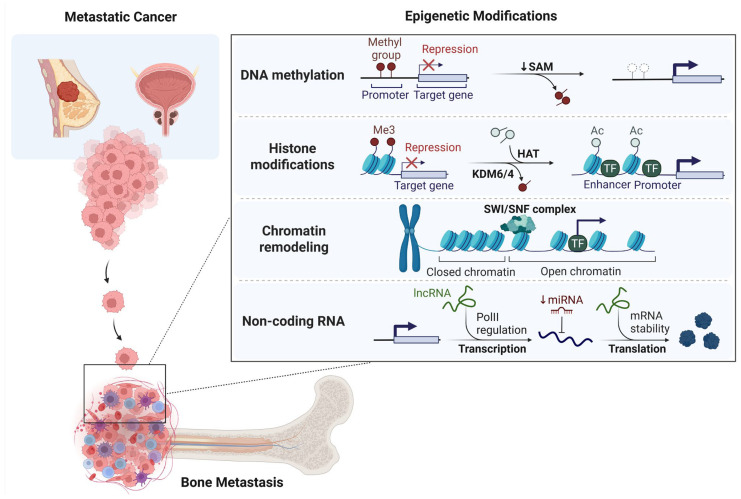
Epigenetic Modifications in Bone Metastasis. This figure illustrates the role of key epigenetic mechanisms in promoting bone metastasis from primary breast and prostate cancers. Tumor cells disseminate from the primary site, enter the circulation, and colonize the bone microenvironment, where epigenetic alterations support their survival and progression. DNA methylation leads to transcriptional silencing by adding methyl groups to promoter regions, often resulting in the downregulation of tumor suppressor genes. Decreased S-adenosylmethionine (SAM) levels contribute to abnormal methylation patterns. Histone modifications, such as methylation and acetylation, regulate chromatin structure and gene expression through the activity of enzymes like histone acetyltransferases (HATs) and demethylases (e.g., KDM6/4). Chromatin remodeling, mediated by complexes such as SWI/SNF, alters nucleosome positioning to shift chromatin between closed (inactive) and open (active) states, thereby controlling the access of transcription factors to DNA. Non-coding RNAs, including long non-coding RNAs (lncRNAs) and microRNAs (miRNAs), further regulate gene expression by modulating transcriptional machinery, mRNA stability, and translation. Together, these epigenetic modifications reprogram gene expression to enhance metastatic potential and adaptation of cancer cells within the bone niche. Created in BioRender. Zhra, M. (2025) https://BioRender.com/0tstbhy (accessed on 27 July 2025).

Growing evidence highlights the crucial role of epigenetic mechanisms in controlling the complex relationships between metastatic cancer cells and the bone microenvironment. These dynamic and reversible modifications, including DNA methylation, histone modifications, non-coding RNAs, and chromatin remodeling, enable tumor cells to adapt, persist, and proliferate within the bone niche. This section delves into the various epigenetic processes that drive metastatic progression, influence niche remodeling, and contribute to therapeutic resistance in bone metastasis. The key epigenetic mechanisms orchestrating skeletal colonization are summarized in Figure 1.

### 2.1. DNA Methylation and Bone Metastasis

DNA methylation is a crucial epigenetic mechanism involved in gene regulation and the maintenance of genome stability in eukaryotes [22]. It primarily occurs at cytosine residues, resulting in the formation of 5-methylcytosine (5mC), and plays a key role in transcriptional silencing, particularly in promoter regions rich in CpG islands [23].

In cancer, DNA methylation is critically implicated in tumor progression and the development of bone metastases. Specifically, methylation of CpG islands within gene promoter regions can silence tumor suppressor genes, thereby altering gene expression and compromising genomic stability [9]. This mechanism is particularly significant in bone metastasis, where hypermethylation of specific genes can enhance the metastatic potential of cancer cells [24].

Cancer cells often exhibit abnormal DNA methylation patterns, characterized by global hypomethylation and localized hypermethylation of CpG islands [25]. These changes contribute to metastasis by silencing key regulatory genes through hypermethylation and activating pro-metastatic genes through hypomethylation [26]. Furthermore, global hypomethylation is associated with chromosomal instability, increased tumor heterogeneity, and poor prognosis across various cancer types [27].

In the context of bone metastasis, aberrant DNA methylation patterns have been observed in several genes. For instance, genes such as *HIN-1*, *RARβ*, *RASSF1A*, *CDH13*, and *RIL* exhibit higher methylation frequencies in metastatic tissues compared to primary tumors [28,29]. Hypermethylation of *HIN-1* (Highly Inhibited in Neoplastic Cells 1), *RAR-β* (Retinoic Acid Receptor Beta), and *RASSF1A* (Ras Association Domain Family Member 1A) is significantly elevated in breast cancer metastases to the bone, brain, and lung, suggesting their potential as biomarkers for metastatic progression [28,30]. Additionally, *CDH13* (Cadherin 13), a gene involved in cell adhesion, shows aberrant methylation correlating with increased invasiveness and poorer clinical outcomes in metastatic breast cancer [29]. *RIL* (Ras-Interaction Protein), implicated in the Ras signaling pathway, exhibits hypermethylation in metastatic tissues compared to primary tumors, suggesting it influences metastatic processes [29].

Furthermore, *TIMP3* (Tissue Inhibitor of Metalloproteinases-3) is frequently silenced by promoter hypermethylation in various types of cancer. The silencing of *TIMP3* leads to increased matrix metalloproteinase (MMP) activity, facilitating extracellular matrix degradation, promoting epithelial–mesenchymal transition (EMT), and enhancing tumor invasion and metastasis [31,32,33]. Collectively, the hypermethylation of these genes contributes to the epigenetic landscape that promotes bone metastasis in different cancers, offering potential avenues for prognostic assessment and therapeutic intervention.

Conversely, promoter hypomethylation has been observed in cancers that metastasize to the bone. For example, hypomethylation of the *ANO1* promoter leads to upregulation of this chloride channel, thereby enhancing prostate cancer cell invasion and bone metastasis [24]. Inhibition or silencing of *ANO1* suppresses tumor growth, induces apoptosis, and reduces metastasis in both prostate and lung cancer models [34,35,36].

DNA methylation significantly influences the bone microenvironment during cancer metastasis, particularly by modulating osteoclast and osteoblast activity [37]. Hypomethylation of genes, such as SOST, which encodes sclerostin, leads to increased sclerostin expression, inhibiting osteoblast differentiation and bone formation by acting as a negative regulator in the Wnt signaling pathway. Conversely, hypermethylation of these genes can reduce sclerostin expression, potentially enhancing osteoblast differentiation and bone formation [38]. In breast cancer bone metastasis, elevated SOST expression correlates with increased metastasis and poorer survival; silencing SOST reduces cell proliferation and migration, while SOST enhances tumor growth through interactions with STAT3 and TGF-β/KRAS pathways; targeting the SOST-STAT3 interaction with the compound S6 shows promise as a therapeutic strategy to inhibit bone metastasis [39].

The RANK/RANKL/OPG signaling pathway plays a pivotal role in bone remodeling, osteoclast differentiation, and activity, as well as bone metastasis [40]. This pathway regulates the formation and function of osteoclasts and is critically involved in the development of bone metastases in several cancers, including breast, prostate, and lung cancers [41,42,43]. *RANKL* (Receptor Activator of Nuclear Factor Kappa-B Ligand) promotes osteoclastogenesis, while OPG (osteoprotegerin) acts as a decoy receptor for RANKL, so their regulation is critical for maintaining bone homeostasis [44]. Moreover, elevated expression of *RANKL* and an increased RANKL:OPG ratio are associated with a higher risk of bone metastases [42,45]. Epigenetic regulation through DNA methylation of CpG islands near the transcription start sites of the *RANKL* and *OPG* genes represses their expression in human osteoblastic cells. Treatment with the demethylating agent 5-azadeoxycytidine significantly increases their mRNA expression, indicating that DNA methylation plays a regulatory role in osteoclastogenesis and bone remodeling [46].

Additionally, DNA methylation can inhibit osteoblast differentiation by silencing genes essential for osteoblast function, such as *RUNX2*, a master transcription factor whose promoter hypermethylation has been associated with reduced osteogenic activity [47]. RUNX2 is essential for breast cancer bone metastasis by enhancing osteotropism and colonization [48]. It promotes integrin α5-mediated adhesion to bone and regulates genes like *ITGBL1* that activate TGFβ signaling [49]. RUNX2 also facilitates the release of extracellular vesicles that induce a pre-metastatic niche in bone [50]. Targeting RUNX2 and its effectors, such as integrin α5, may provide therapeutic opportunities for bone metastasis [51].

Understanding these epigenetic mechanisms is crucial for developing targeted therapies for bone metastasis, as the imbalance between bone resorption and formation facilitates the establishment and progression of metastatic lesions in the bone.

Demethylation of gene promoters can reactivate silenced genes, potentially reversing the metastatic phenotype [52]. This process is mediated by enzymes such as TET proteins, which convert 5-methylcytosine (5mC) to 5-hydroxymethylcytosine (5hmC), resulting in either passive or active demethylation [53]. In the context of bone metastasis, demethylation can influence the expression of genes involved in bone remodeling and metastasis, such as those regulating osteoclast and osteoblast activity [54]. Targeting demethylation pathways offers potential therapeutic strategies. For instance, inhibiting TET enzymes has been shown to modulate the epithelial–mesenchymal transition (EMT) process, a critical step in metastasis [55]. Drugs like decitabine, a DNA methyltransferase inhibitor, have been explored for their ability to demethylate and reactivate tumor suppressor genes, thereby inhibiting metastasis [56].

### 2.2. Histone Modifications and Bone Metastasis

Histone modifications play a crucial role in regulating chromatin structure and function, thereby significantly impacting processes such as transcription, DNA repair, and replication. Key types of histone modifications include acetylation, methylation, phosphorylation, and ubiquitination, which occur on specific residues of histone tails [57]. These modifications can alter chromatin accessibility and stability, creating interaction surfaces for other proteins [58]. Histones form the core structural component of nucleosomes, the basic unit of chromatin organization. Each nucleosome consists of approximately 147 base pairs of DNA wrapped around an octameric core of histones H2A, H2B, H3, and H4 [59]. These histone proteins contain flexible N-terminal tails that extend from the nucleosome core and are subject to various post-translational modifications (PTMs) [60].

#### 2.2.1. Histone Acetylation

Histone acetylation is a post-translational modification (PTM) that involves the addition of acetyl groups to lysine residues in histone tails, a reaction catalyzed by histone acetyltransferases (HATs), while histone deacetylases (HDACs) remove these acetyl groups [61]. This reversible modification plays a critical role in regulating chromatin structure and gene expression. Acetylation by HATs neutralizes the positive charge of lysines, leading to a relaxed chromatin state (euchromatin) that aids transcription [62]. Conversely, HDACs remove these acetyl groups, restoring the positive charge and resulting in chromatin condensation (heterochromatin), thereby suppressing transcription [63]. Proper balance between HAT and HDAC activity is essential for maintaining normal cellular function. This dynamic interaction influences essential vital cellular processes such as stem cell identity, differentiation, and tumorigenesis [64]. Dysregulation of HATs and HDACs has been involved in various diseases, including cancer, making them important targets for therapeutic intervention [65].

The bone microenvironment is rich in transforming growth factor-beta (TGF-β), a key mediator of bone metastasis, especially in prostate and breast cancers [66,67]. TGF-β promotes the acetylation of Krüppel-like factor 5 (KLF5) at lysine 369 (K369), resulting in the formation of acetylated KLF5 (Ac-KLF5) [68]. Ac-KLF5 subsequently activates downstream targets such as CXCR4 and IL-11, which stimulate osteoclastogenesis and support the metastatic colonization of bone [68]. Beyond its role in bone remodeling, Ac-KLF5 also drives epithelial–mesenchymal transition (EMT), enhancing tumor cell invasiveness and resistance to chemotherapeutic agents like docetaxel [69,70]. This establishes a vicious cycle of tumor proliferation and bone degradation [68]. Targeting the TGF-β/Ac-KLF5 signaling axis presents a promising therapeutic strategy, with agents such as nitazoxanide demonstrating efficacy in preclinical models by inhibiting Ac-KLF5-mediated bone metastasis [71].

#### 2.2.2. Histone Methylation

Histone methylation is an important epigenetic modification that involves the addition of methyl groups to specific amino acids, primarily lysine (K) and arginine (R), on histone proteins, particularly histones H3 and H4. This process is catalyzed by histone methyltransferases (HMTs), while histone demethylases (HDMs) are responsible for removing these methyl groups [72]. Histone methylation can activate or repress transcription, influencing various biological processes and potentially contributing to tumor development when dysregulated [73].

Histone methylation plays a pivotal role in regulating the epithelial–mesenchymal transition (EMT) and facilitating bone metastasis in various cancers, particularly prostate cancer. The histone demethylase KDM5D (JARID1D) specifically demethylates H3K4me3, thereby modulating the transcriptional activity of the androgen receptor (AR). This regulation impacts osteoclast differentiation through the RANKL signaling pathway. Disruption of this regulatory mechanism is linked to an increased risk of bone metastasis in prostate cancer patients [74,75]. Other histone demethylases, such as KDM5C and KDM5B, are also implicated in prostate cancer progression by promoting EMT and regulating PI3K/AKT signaling [76,77]. Moreover, KDM4B plays crucial roles in cancer progression and bone homeostasis by regulating osteoclast differentiation and bone remodeling, with its loss linked to skeletal aging and osteoporosis [78,79]. KDM4B is frequently overexpressed in various cancers, where it promotes gene expression under hypoxic conditions and enhances metabolic activity in castration-resistant prostate cancer (CRPC) by functioning as a coactivator of c-Myc [80]. It also contributes to key oncogenic processes such as cell proliferation, survival, and metastasis [81]. In pancreatic cancer, KDM4B regulates the expression of ZEB1 during TGF-β–induced epithelial-to-mesenchymal transition (EMT), and its silencing significantly impairs cancer cell migration, invasion, and EMT [82]. However, some histone demethylases, such as KDM6B (JMJD3), play a dual role in cancer metastasis, highlighting the importance of cellular context [83]. For instance, KDM6B has been shown to inhibit breast cancer metastasis by regulating the Wnt/β-catenin signaling pathway [84], while promoting metastasis in other cancers such as esophageal squamous cell carcinoma and osteosarcoma [85,86].

Furthermore, the histone methyltransferase SETD1A, which catalyzes the methylation of histone H3 at lysine 4 (H3K4), is upregulated in metastatic breast cancer and promotes metastasis by activating matrix metalloproteinase (MMP) expression [87]. This enzyme also enhances estrogen receptor alpha (ERα) target gene expression, thereby promoting cell survival and migration in ER-positive breast cancer [88]. Additionally, histone methyltransferases such as SETDB1, which adds methyl groups to histone H3 at lysine 9 (H3K9), are implicated in breast cancer metastasis [89]. Moreover, SMYD3 promotes cancer invasion by upregulating MMP-9 expression [90].

### 2.3. Chromatin Remodeling and Bone Metastasis

Chromatin remodeling is a dynamic process that modifies chromatin architecture, facilitating access to condensed genomic DNA for regulatory transcription machinery and thereby controlling gene expression [91]. Chromatin remodeling factors like ARID2 and ARID1A, essential components of the SWI/SNF complex, are critical in regulating cancer progression and metastasis. Loss of ARID2 has been associated with increased tumor growth, impaired DNA repair, and heightened metastatic potential [92], while ARID1A deficiency promotes cancer cell proliferation and invasion, including bone metastasis [93]. These findings underscore the significance of chromatin remodelers as potential therapeutic targets for managing bone metastatic disease.

Moreover, BRG1 (SMARCA4), a chromatin remodeling ATPase, is essential for the survival and proliferation of PTEN-deficient prostate cancer cells, as demonstrated by Ding et al. The study indicates that BRG1 also contributes to metastatic potential, with in vivo experiments showing that silencing BRG1 significantly reduces bone colonization and osteolysis in mouse models, highlighting its role in promoting bone metastasis and suggesting it as a potential therapeutic target for inhibiting skeletal metastasis [94].

### 2.4. Non-Coding RNAs and Bone Metastasis

Non-coding RNAs (ncRNAs), including long non-coding RNAs (lncRNAs), microRNAs (miRNAs), and circular RNAs (circRNAs), play crucial roles in regulating bone metastasis across several cancer types, notably prostate, breast, and lung cancer [95,96,97]. These ncRNAs modulate various cellular processes, such as proliferation, invasion, migration, apoptosis, and stemness [98]. They act as regulators of bone metastasis formation and potential biomarkers for cancer diagnosis and prognosis [99]. ncRNAs can function as both tumor suppressors and oncogenes, influencing carcinogenesis through complex interactions [100,101].

#### 2.4.1. Long Non-Coding RNAs (lncRNAs)

Long non-coding RNAs (lncRNAs) are RNA transcripts longer than 200 nucleotides that lack protein-coding potential [102]. They play crucial roles in regulating gene expression at multiple levels, including chromatin remodeling, transcription, and post-transcriptional processing [103]. LncRNAs such as NORAD and MALAT1 play significant roles in the progression of cancer to bone metastasis. MALAT1 plays different roles in bone metastasis, depending on the type of cancer. In non-small cell lung cancer (NSCLC), MALAT1 is overexpressed and enhances tumor cell proliferation, migration, and invasion by activating signaling pathways such as ERK/MAPK and Akt/mTOR, thereby promoting bone metastasis [104,105,106]. Conversely, in melanoma and breast cancer, MALAT1 acts as a suppressor of bone metastasis. It binds to the transcription factor Tead3, preventing the activation of Nfatc1, a key regulator of osteoclast differentiation, thus inhibiting bone resorption and metastatic colonization [107]. These findings underscore the significance of cancer type in determining the role of MALAT1 in bone metastasis.

NORAD also exhibits cancer type-specific roles in bone metastasis. In prostate cancer, NORAD is overexpressed and promotes bone metastasis by enhancing the release of extracellular vehicles (EVs) through the miR-541-3p/PKM2 axis. These EVs are internalized by bone marrow stromal cells, fostering a microenvironment conducive to tumor colonization [108]. Conversely, in breast cancer, NORAD acts as a metastasis suppressor. Its overexpression inhibits cell proliferation, migration, and invasion by binding to miR-155-5p, leading to the upregulation of SOCS1, a suppressor of cytokine signaling [109]. These findings highlight the context-dependent functions of NORAD in cancer progression and bone metastasis.

#### 2.4.2. MicroRNAs (miRNAs)

MicroRNAs (miRNAs) are small non-coding RNAs, approximately 18–24 nucleotides long, which regulate gene expression post-transcriptionally by binding to target messenger RNA (mRNA) [110]. Dysregulation of miRNAs has been implicated in numerous diseases, particularly cancer, where they can act as tumor suppressors or oncogenes [111].

miRNAs play pivotal roles in the development and progression of bone metastases in breast cancer [112]. Specific miRNAs, such as miR-23a-3p, miR-27a-3p, miR-20a-5p, and miR-335-3p, are associated with the earlier onset of bone metastases, suggesting their potential as biomarkers for early detection of this condition. Conversely, miR-30b-3p and miR-139-3p have been linked to a reduced occurrence of bone metastases, indicating a possible protective role [113].

In the bone microenvironment, miRNAs regulate osteoclast activity, a crucial process for bone resorption. For instance, miR-16 enhances osteoclast function and bone destruction in breast cancer bone metastasis, while miR-133a and miR-223 inhibit these processes, highlighting their potential as therapeutic targets [114].

Furthermore, miRNAs contribute to the formation of pre-metastatic niches and modulate the immune response, facilitating cancer cell colonization in bone tissue [115,116]. Their multifaceted roles underscore the importance of miRNAs as both biomarkers and therapeutic targets in managing bone metastases in cancer patients.

#### 2.4.3. Circular RNAs (circRNAs)

Circular RNAs (circRNAs) are a class of non-coding RNAs with a covalently closed loop structure, lacking 5′ caps and 3’ poly(A) tails [117]. CircRNAs function as microRNA sponges, RNA-binding protein regulators, and transcriptional modulators [118,119]. CircRNAs are emerging as crucial regulators in bone metastasis and cancer progression. For instance, circIKBKB promotes breast cancer bone metastasis by activating NF-κB signaling, which upregulates bone remodeling factors such as RANKL, and PTHrP [120,121]. These factors enhance osteoclastogenesis and bone resorption, facilitating the establishment of a bone microenvironment conducive to tumor colonization [120,121]. Targeting circIKBKB or its regulatory pathways, such as EIF4A3, may offer potential therapeutic strategies for preventing or treating bone metastases in breast cancer patients [120]. Moreover, in non-small cell lung cancer (NSCLC), circ_0060937 has been reported to be significantly upregulated in patients with bone metastasis. Its expression correlates with poor prognosis and advanced clinical features, suggesting its potential as a biomarker of bone metastasis in NSCLC [122].

Furthermore, circular RNA circMMP2(6,7) has been identified as significantly upregulated in breast cancer tissues with bone metastasis [123]. This upregulation is associated with increased metastatic potential. Mechanistically, circMMP2(6,7) binds to the promoters of bone-remodeling genes S100A4 and LGALS3, forming a complex with β-catenin and PRMT5. This complex induces specific histone modifications (H3R2me1 and H3R2me2s), leading to the activation of transcription and disruption of bone homeostasis. These changes facilitate the formation of a bone metastatic niche that supports cancer progression. Importantly, treatment with GSK591, a selective PRMT5 inhibitor, effectively inhibits the circMMP2(6,7)/β-catenin/PRMT5 complex-induced bone metastasis, highlighting a potential therapeutic target for managing breast cancer bone metastasis [123].

#### 2.4.4. Epitranscriptomic Modifications

N6-methyladenosine (m^6^A) has emerged as a critical epitranscriptomic modification regulating RNA metabolism and gene expression in cancer progression and metastasis [124]. m^6^A modifications are catalyzed by “writers” such as METTL3 and METTL14, removed by “erasers” like FTO and ALKBH5, and interpreted by “readers” such as YTH domain-containing proteins [125,126]. In metastatic breast and prostate cancers, dysregulated m^6^A marks influence the stability, splicing, and translation of non-coding RNAs and mRNAs that control tumor cell plasticity and interaction with the bone microenvironment [127,128].

For instance, in prostate cancer, METTL3-mediated m^6^A modification increases the stability of the long non-coding RNA PCAT6. The m^6^A-marked PCAT6 is recognized by the reader protein IGF2BP2, which in turn stabilizes IGF1R mRNA. This upregulation of IGF1R activates the IGF signaling pathway, thereby promoting bone metastasis. Functionally, disruption of the PCAT6–IGF2BP2–IGF1R axis reduces the bone metastatic potential of prostate cancer cells both in vitro and in vivo [127].

Another example is breast cancer, where the m^6^A reader protein YTHDF1 promotes osteolytic bone metastasis by enhancing the translation of EZH2 and CDH11. By binding to m^6^A-modified mRNAs, YTHDF1 increases their protein expression, facilitating cancer cell migration, invasion, and bone degradation. Knockdown of YTHDF1 in vivo significantly reduces bone metastasis and osteolytic lesions, highlighting its potential as a therapeutic target [128].

Furthermore, m^6^A modification critically regulates osteoblast and osteoclast differentiation by modulating key signaling pathways such as Wnt/β-catenin and RANKL/RANK. For instance, the m^6^A “writer” METTL3 promotes osteoblast differentiation, while the “eraser” FTO influences osteoclast maturation. This epitranscriptomic regulation is essential for maintaining bone homeostasis, and presents potential therapeutic targets for bone disease [129].

### 2.5. Epigenetic Interactions with Bone-Resident Cells

Bone metastasis occurs within a uniquely specialized microenvironment, where epigenetic mechanisms interact intricately with bone-resident cells, including osteoblasts, osteoclasts, and stromal cells. These interactions are fundamentally distinct from the generic metastatic processes observed in soft tissues and are critical in shaping tumor behavior, bone remodeling, and therapeutic response.

One of the key epigenetic alterations involved in this process is DNA methylation, which plays a significant role in regulating the balance between osteoprotegerin (OPG) and receptor activator of nuclear factor-κB ligand (RANKL), two central mediators of osteoclastogenesis [130]. Aberrant methylation patterns can disrupt this balance; for instance, hypermethylation may silence the *OPG* gene, thereby promoting osteoclast activation and bone resorption, while hypomethylation of osteolytic genes such as *RANKL* may enhance bone degradation and tumor-induced osteolysis [54]. In prostate cancer bone metastases, distinct promoter methylation signatures have been identified in genes such as *SFRP2*. These epigenetic modifications alter gene expression, disrupting differentiation pathways and deregulating Wnt signaling, thereby promoting an osteoblast-like phenotype in tumor cells that enhances osteomimicry and facilitates colonization of the bone microenvironment [131].

Histone modifications further contribute to the regulation of bone-resident cell function during metastasis. In particular, the expression of NFATc1, a master regulator of osteoclast differentiation, is epigenetically modulated through demethylation of the repressive H3K27me3 histone mark [132]. Additionally, osteogenic differentiation involves distinct chromatin remodeling events, characterized by increased acetylation of histones H3 and H4, alongside reduced trimethylation at H3K9 and H3K27 [133]. Enzymes such as histone deacetylases (HDACs) and histone acetyltransferases (HATs) orchestrate these changes, influencing not only the differentiation of osteoblasts and osteoclasts, but also their responsiveness to tumor-derived signals in the bone microenvironment [134].

Non-coding RNAs, particularly microRNAs (miRNAs), play a crucial role in mediating the crosstalk between cancer cells and bone-resident cells. Several miRNAs involved in normal bone remodeling become dysregulated in bone metastasis [135]. For instance, miR-21 has been shown to promote osteoblastic activity and mineralization under physiological conditions [136], but also contributes to bone resorption by enhancing osteoclast differentiation [137]. Furthermore, depletion of miR-15 and miR-16, combined with the upregulation of miR-21, activates TGF-β signaling in prostate cancer cells, thereby facilitating bone marrow colonization and osteolytic activity [138]. In addition, miR-494-3p has been shown to promote osteolytic bone metastasis in breast cancer by simultaneously enhancing osteoclastogenesis and suppressing osteoblast formation [139]. Similarly, miR-152-3p contributes to osteolytic progression in prostate cancer bone metastasis by promoting bone resorption [140]. Importantly, bone marrow stromal cells can release miRNAs that influence not only bone homeostasis but also the colonization and adaptation of metastatic tumor cells to the bone niche [141].

Altogether, these findings underscore the dynamic and reciprocal epigenetic regulation between cancer cells and the bone microenvironment. Bone metastasis involves specialized molecular interactions that reshape this environment. Insights into how DNA methylation, histone modifications, and non-coding RNAs influence osteoblasts, osteoclasts, and stromal components present promising avenues for targeted therapies.

### 2.6. Epigenetic Modulation of Bone Homeostasis

Bone epigenetic mechanisms play a crucial role in the interactions between osteoblasts, osteoclasts, and bone marrow stromal cells (BMSCs) within the bone microenvironment. This interaction is essential in maintaining bone homeostasis and is significantly influenced by various epigenetic modifiers. Evidence suggests that epigenetic modifications, including DNA methylation, histone modifications, and non-coding RNA expression, can significantly impact osteoclastogenesis, osteoblast suppression, and tumor–bone cell interactions.

Osteoclastogenesis is notably influenced by epigenetic regulators that modulate the differentiation of osteoclast precursors. For instance, studies have demonstrated that RANKL is critical for osteoclast differentiation and is regulated by epigenetic factors such as DNA methylation and histone acetylation, which alter gene expression patterns necessary for this process [142,143]. Additionally, intercellular adhesion molecules contribute to the interaction between BMSCs and hematopoietic cells, participating in the development and maturation of osteoclasts. The modulation of these interactions through epigenetic changes can either promote or inhibit osteoclast function, depending on the environmental cues present within the bone microenvironment [144,145].

In terms of osteoblast suppression, multiple myeloma (MM) serves as a pertinent example. Adamik et al. highlighted the role of epigenetic changes, such as aberrant DNA methylation and altered expression of non-coding RNAs, in suppressing osteoblast function in MM. This suppression is critical in the pathogenesis of bone disease associated with the disorder, where the microenvironment exhibits alterations that inhibit bone formation by osteoblasts [142]. Additionally, RUNX2, a key transcription factor in osteoblast differentiation, is regulated epigenetically, influencing the expression of crucial genes associated with osteoblast maturation and function [146,147]. Disruption of these epigenetic marks can result in defects in bone formation, further compounded by tumor-secreted factors that alter the behavior of osteoblasts and inhibit their activity [49].

Furthermore, tumor–bone cell crosstalk underlies a complex interplay in which malignant cells can modify the bone microenvironment, thereby promoting their survival and proliferation. Tumors such as breast cancer have shown the ability to utilize the bone microenvironment through osteomimetic properties, allowing them to evade immune surveillance in the bone niche [49,148]. Kim et al. demonstrated that CrkII functions as a bifunctional protein, both regulating osteoclast differentiation and negatively influencing osteoblast activity, thereby highlighting the delicate balance maintained between these cell types under the influence of epigenetic factors [149]. The shaping of the bone microenvironment through epigenetic changes ultimately facilitates the presence and metastasis of tumor cells [150].

The interactions within the bone microenvironment, particularly between osteoblasts, osteoclasts, and BMSCs, are intricately regulated by epigenetic mechanisms. These mechanisms directly impact osteoclastogenesis, osteoblast activity, and the malignant potential of tumors in the bone, highlighting the importance of epigenetic modifiers in bone biology.

### 2.7. Epigenetics of Dormancy and Lesion Heterogeneity

The current literature indicates that there is no consensus on which specific epigenetic marks are solely responsible for driving tumor cell dormancy or reactivation in bone metastases. Many studies have identified various epigenetic modifications that can influence these processes; however, the diversity of cancer types and the complexity of the bone microenvironment mean that no single epigenetic mark has yet emerged as a universal driver of these processes.

Several studies have identified epigenetic regulation as a key factor in controlling dormancy. For instance, *SPARC* promoter methylation has been linked to maintaining dormancy in prostate cancer cells within the bone microenvironment, indicating that DNA methylation may function as an important epigenetic marker [151]. Similarly, the orphan nuclear receptor *NR2F1* is epigenetically upregulated in dormancy models across various cancer types, connecting retinoic acid signaling and DNA demethylation to the initiation and sustenance of dormancy [152]. These findings highlight the fact that changes in DNA methylation and related alterations in gene expression are central to the dormancy phenotype.

Reviews of the wider dormancy mechanism in bone metastases highlight the fact that, although epigenetic reprogramming is clearly involved, these modifications occur alongside a variety of other signaling pathways and microenvironmental factors. For example, the interaction between disseminated tumor cells (DTCs) and bone marrow niches, including signals from stromal cells and cytokines, further influences the epigenetic landscape of these cells [153,154]. Therefore, combining epigenetic changes with pathways such as p38MAPK/ERK balance is essential, as shown in dormancy models [155]. Ultimately, a single epigenetic marker is unlikely to be the only factor driving the switch between dormancy and reactivation.

The complexity is increased by the fact that epigenetic modifications may also influence other related aspects, such as drug resistance in bone metastases [156]. Although this observation highlights the importance of epigenetics in the metastatic cascade, it also emphasizes that different epigenetic marks could be relevant at various stages of the metastatic process. Consequently, emerging evidence suggests that although several candidate markers, such as *SPARC* promoter methylation and *NR2F1* upregulation, are significant, they do not define a single, universal driver of dormancy versus reactivation.

Although various epigenetic modifications have been linked to the dormancy and subsequent reactivation of tumor cells in bone metastases, the lack of consistent findings across studies [151,152,153,154,155] suggests that a consensus has not yet been reached. Future research must continue to unravel the intricate relationships between epigenetic changes and the broader signaling networks of the bone microenvironment, to establish definitive mechanistic markers for these processes.

Although substantial evidence shows that dynamic changes in signaling pathways (including Wnt/β-catenin and its inhibitors) drive the divergent osteolytic versus osteoblastic responses [157,158], the literature lacks direct, integrative epigenomic studies to determine whether unique epigenetic patterns accompany these functional differences. This represents a significant gap in current research, necessitating future studies that incorporate genome-wide epigenetic profiling to determine whether specific epigenetic modifications serve as markers or drivers of the distinct lesion phenotypes observed in bone metastases.

**Figure 2:**
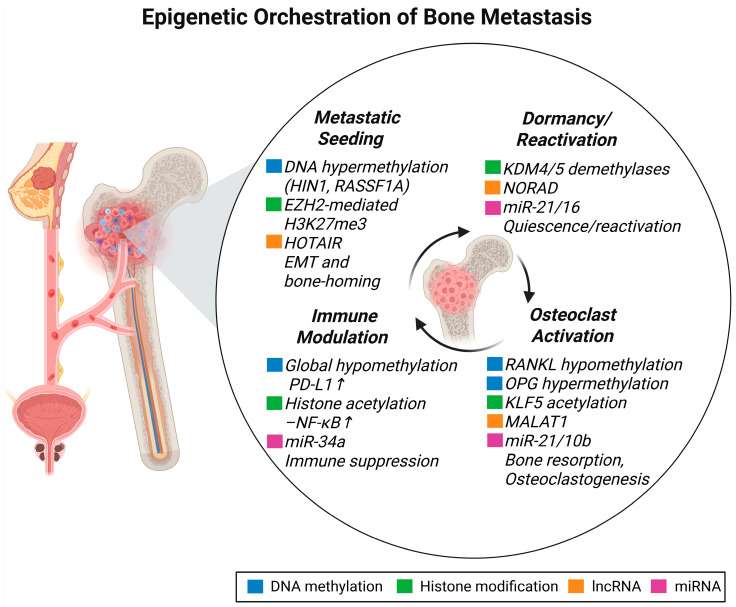
Epigenetic Orchestration of Bone Metastasis. This figure illustrates the epigenetic orchestration of bone metastasis, focusing on four interconnected processes governed by DNA methylation, histone modifications, long non-coding RNAs (lncRNAs), and microRNAs (miRNAs). In the metastatic seeding phase, DNA hypermethylation of tumor suppressors such as *HIN1* and *RASSF1A*, along with EZH2-mediated H3K27 trimethylation and lncRNA HOTAIR, facilitates epithelial-to-mesenchymal transition (EMT) and bone colonization. Tumor dormancy and reactivation are regulated by histone demethylases (KDM4/5), lncRNA NORAD, and miRNAs such as miR-21 and miR-16. During osteoclast activation, *RANKL* hypomethylation, *OPG* hypermethylation, KLF5 acetylation, lncRNA MALAT1, and miRNAs like miR-21 and miR-10b promote osteolytic activity. Immune evasion is driven by global DNA hypomethylation, PD-L1 upregulation, histone acetylation (enhancing NF-κB activity), and miR-34a-mediated suppression of immune responses. Together, these epigenetic mechanisms coordinate the progression of bone metastasis. Created in BioRender. Zhra, M. (2025) https://BioRender.com/0tstbhy (accessed on 27 July 2025).

Collectively, understanding the epigenetic landscape of bone metastasis not only elucidates the mechanisms underlying tumor progression, but also provides valuable biomarkers for monitoring metastatic spread and paves the way for the development of targeted epigenetic therapies (Figure 2).

## 3. Epigenetic Therapies and Clinical Implications

Bisphosphonates, denosumab, and other anti-resorptive agents are frequently employed in the treatment of bone metastases associated with various cancers; however, they have not shown substantial benefits in reducing patient morbidity or mortality [159]. Consequently, it is essential to discover new therapeutic targets [159]. Considering the current insights into the intricate relationships between the tumor microenvironment (TME) and the cancer epigenome, a diverse array of epigenome-modifying agents have been investigated for their therapeutic efficacy [160]. These agents encompass DNA methyltransferase (DNMT) inhibitors, which counteract abnormal DNA hypermethylation; histone deacetylase (HDAC) inhibitors, which facilitate the restoration of gene expression through histone acetylation; and histone methyltransferases such as EZH2 and DOT1L, which are often found to be dysregulated in various cancers. Furthermore, inhibitors that target embryonic ectoderm development (EED), a fundamental element of the Polycomb repressive complex 2 (PRC2), have demonstrated potential in altering chromatin configurations [161,162]. Bromodomain and extra-terminal domain (BET) inhibitors, including BRD4 antagonists, disrupt the recognition of acetylated histones and inhibit oncogenic transcriptional programs [162]. Additional emerging classes of agents include histone demethylase inhibitors (KDM inhibitors), which address epigenetic changes induced by oncometabolites [163]. These therapeutic agents not only address intrinsic epigenetic dysregulation within tumors, but also affect the immunogenic properties and cellular makeup of the TME, thereby supporting their incorporation into combination therapies alongside immunotherapeutic and cytotoxic treatments [160,164].

### 3.1. DNA Methylation Inhibitors

DNA methyltransferases (DNMTs) are pivotal enzymes in epigenetic regulation, comprising three catalytically active forms: DNMT1, which maintains DNA methylation during replication, and DNMT3A and DNMT3B, which mediate de novo methylation [165]. DNMT inhibitors (DNMTis), also known as hypomethylating agents (HMAs), are classified into nucleoside analogs and non-nucleoside inhibitors [165].

The nucleoside analogs 5-azacytidine and decitabine, which have received FDA approval, are derivatives of cytidine that incorporate into DNA (in the case of decitabine) or into both DNA and RNA (in the case of 5-azacytidine) [161]. This incorporation results in the formation of covalent adducts with DNA methyltransferase (DNMT) during the replication process [165]. Consequently, this leads to a reduction in DNMT enzyme levels, promoting passive demethylation, which in turn reactivates tumor suppressor genes, inhibits cellular proliferation, and induces differentiation or apoptosis in cancerous cells. Both agents are clinically sanctioned for the treatment of myelodysplastic syndromes (MDSs) and acute myeloid leukemia (AML) [166,167].

In the context of solid tumors, decitabine has demonstrated the ability to upregulate immune-related gene expression, such as that of major histocompatibility complex (MHC) molecules and cytokines [168]. This upregulation may augment tumor immunogenicity and enhance the effectiveness of immunotherapeutic approaches [168]. A phase I clinical trial examining the combination of decitabine with immune checkpoint inhibitors in patients suffering from advanced solid tumors revealed an increase in the expression of immune-related genes and a greater infiltration of immune cells within the tumor microenvironment, indicating a potential synergistic interaction between epigenetic therapy and immunotherapy [169]. The administration of decitabine has been linked to the elicitation of a viral mimicry response within cancer cells [170]. This phenomenon entails the activation of endogenous retroviral components, resulting in the synthesis of double-stranded RNA and the subsequent activation of interferon signaling pathways [170]. These immunostimulatory effects may enhance the susceptibility of tumors to immune-mediated eradication [170]. Comparative studies suggest that decitabine is more potent in inducing DNA hypomethylation, while 5-azacytidine exerts broader epigenetic effects, due to its incorporation into both DNA and RNA [171]. This dual incorporation allows 5-azacytidine to affect RNA processing and protein synthesis, contributing to its antineoplastic activity [170].

In bone metastasis models, DNA methylation inhibitors have demonstrated both therapeutic potential and mechanistic complexity [172]. Ateeq et al. showed that 5-aza-2′-deoxycytidine (5-aza-CdR) reactivated the tumor suppressor *RASSF1A* and inhibited tumor growth in non-invasive breast cancer cells, but simultaneously upregulated prometastatic genes (*uPA*, *CXCR4*, *heparanase*, *SNCG*, *VEGF*, *TGF-β*) through promoter demethylation, enhancing invasion and migration both in vitro and in vivo [172]. Decitabine has been shown to downregulate RANK and inhibit NF-κB signaling, thereby limiting osteoclast differentiation and bone resorption—key processes in the establishment of skeletal metastases [173]. Similarly, azacitidine has been reported to reduce bone and brain metastases by inducing apoptosis and suppressing Wnt signaling, resulting in decreased metastatic burden and improved survival in vivo [174,175]. Together, these findings suggest that, despite potential context-dependent risks, DNMT inhibitors such as decitabine and azacitidine can favorably modulate the bone microenvironment and tumor–stroma interactions, offering a promising epigenetic approach to impede skeletal colonization and progression.

To address these challenges, second-generation agents such as guadecitabine (SGI-110), a dinucleotide formed by linking decitabine to deoxyguanosine, have been developed to improve metabolic stability and extend in vivo exposure [175]. Guadecitabine has shown encouraging results in clinical trials, including a phase I study where it was administered alongside pembrolizumab in patients with advanced solid tumors, revealing a favorable safety profile and potential for immunomodulatory synergy [176]. Yet, outcomes specific to bone metastases remain lacking, underlining the need for skeletal endpoints in future trials.

More recently, GSK3685032 is a groundbreaking non-nucleoside, reversible inhibitor of DNA methyltransferase 1 (DNMT1) that specifically targets the maintenance methylation process without integrating into DNA or causing DNA damage [177]. In contrast to conventional nucleoside analogs like decitabine and azacitidine, which necessitate incorporation into replicating DNA and may lead to cytotoxic effects, GSK3685032 interacts non-covalently with the CXXC domain and the target recognition domain (TRD) of DNMT1 [178]. This interaction triggers conformational alterations that hinder DNMT1′s ability to recognize hemi methylated DNA during the S-phase of the cell cycle, thus selectively inhibiting maintenance methylation [179]. This unique mechanism enables GSK3685032 to operate independently of DNA replication, presenting a more targeted and potentially safer therapeutic option [179]. Preclinical investigations have shown that GSK3685032 effectively induces significant DNA hypomethylation, reactivates silenced tumor suppressor genes, and suppresses cancer cell growth in vitro [177]. In murine acute myeloid leukemia (AML), GSK3685032 demonstrated enhanced tumor regression and survival advantages over decitabine, alongside better tolerability and diminished hematologic toxicity [177]. However, its potential impact on the hypoxic, slow-cycling bone microenvironment, where nucleoside analogs often fail, remains unexplored, warranting future preclinical investigations [177].

Combining DNMTis with other epigenetic or immune-based therapies represents a promising direction [160]. For example, low-dose azacitidine combined with the HDAC inhibitor Entinostat achieved durable responses in a phase I/II trial in metastatic non-small cell lung cancer [180]. Ongoing research aims to optimize such regimens, particularly for hard-to-treat metastatic niches, such as bone. To maximize their therapeutic utility in bone metastasis, future clinical trials must incorporate bone-specific pharmacokinetic assessments, histopathological analyses, and bone-targeted outcome measures. Furthermore, integrating epigenetic biomarkers such as CXCR4, RANKL, or Wwox methylation status may enhance patient stratification and therapeutic precision.

### 3.2. Histone Deacetylase Inhibitors

Histone deacetylase inhibitors (HDACis) represent a diverse class of epigenetic therapies that can be classified into hydroxamic acids (such as vorinostat, panobinostat, and belinostat), cyclic peptides (like romidepsin), benzamides (including entinostat and mocetinostat), and aliphatic acids (including valproic acid and phenylbutyrate) [181,182]. These compounds demonstrate anticancer properties by preventing the deacetylation of lysine residues on both histone and non-histone proteins, which in turn reactivates tumor suppressor genes and triggers apoptosis, differentiation, and cell cycle arrest [182]. Although numerous HDACis have demonstrated clinical efficacy in hematologic cancers, their application to solid tumors, especially in the context of bone metastasis, poses distinct challenges [181].

For instance, romidepsin and belinostat, approved for peripheral T-cell lymphoma, and chidamide, used in China for PTCL and under study for breast cancer, have shown antitumor activity, but lack robust data in bone metastasis [183,184]. Etinostat, though showing limited efficacy as a monotherapy in solid tumors, has demonstrated significant improvement in progression-free survival when combined with exemestane in patients with hormone receptor-positive, HER2-negative advanced breast cancer [185]. REC-2282 (AR-42), an oral HDACi in early trials for glioblastoma and neuroendocrine tumors, shows promise in resistant cancers, though its role in bone metastasis is unclear [186].

Givinostat, another HDACi currently approved for Duchenne muscular dystrophy, has also demonstrated anti-inflammatory and anti-proliferative effects and is being investigated in myeloproliferative neoplasms, including polycythemia vera, where its potential role in modulating bone marrow pathology may have relevance to bone-involved malignancies [187]. Abexinostat, a hydroxamic acid HDACi, has shown partial and complete responses in patients with relapsed lymphoma, and is currently being studied in solid tumors; however, its efficacy in bone metastases has yet to be elucidated [188].

Several HDACis also modulate non-histone targets and signaling pathways, including NF-κB and p53, contributing to their broader cytotoxic and immunomodulatory effects [189]. To improve therapeutic specificity and safety, isoform-selective HDAC inhibitors are under development [84,190]. Tubastatin A, a selective HDAC6 inhibitor, disrupts cytoskeletal dynamics and has shown potential in reducing metastatic behavior, while PCI-34051, which selectively targets HDAC8, is being evaluated for its immune-modulatory and anti-tumor properties in specific cancer types [84,190].

The bone microenvironment poses unique challenges for HDACi therapy, due to its highly specialized cellular composition and signaling milieu. Factors such as TGF-β, IL-6, and bone-derived growth factors released during osteolysis can alter HDACi response by promoting survival signaling and epigenetic plasticity in metastatic cells [191,192]. Additionally, the dense extracellular matrix and hypoxic niches in bone may impede drug penetration and influence HDAC target accessibility, resulting in reduced efficacy or unintended activation of pro-metastatic pathways [191]. Unlike other tissues where HDACis primarily exert antitumor effects by reactivating silenced tumor suppressors, the bone microenvironment introduces additional complexity [192]. The release of growth factors like TGF-β and IGF-1 during osteolysis promotes cancer cell survival and epigenetic adaptation, potentially reducing HDACi efficacy or triggering pro-metastatic gene programs [67]. Furthermore, the dense extracellular matrix and localized hypoxia can impair drug penetration and alter histone modification dynamics, influencing HDAC target accessibility and therapeutic outcomes [67,159,192].

Despite active research, the clinical application of HDACis in bone metastasis remains limited by several challenges, including inconsistent drug bioavailability in bone tissue, off-target effects on bone-resorbing and bone-forming cells, and a lack of bone-specific biomarkers to guide treatment response [191]. Emerging strategies to overcome these barriers include co-administration with bisphosphonates, RANKL inhibitors, immunotherapies, and DNMT inhibitors to mitigate pro-osteolytic effects and enhance antitumor activity [180,192]. Advancing HDACi use in bone metastasis will require the integration of skeletal-focused endpoints, improved delivery systems targeting bone tissue, and rationally designed combination regimens tailored to the unique dynamics of the bone microenvironment [192].

### 3.3. Histone Methyltransferase and Demethylase Inhibitors

Histone methyltransferases (HMTs) and demethylases (HDMs) regulate chromatin accessibility and gene expression by catalyzing the addition or removal of methyl groups on specific lysine or arginine residues of histone tails [193]. Among HMTs, histone lysine methyltransferases (KMTs) like EZH2, DOT1L, G9a (EHMT2), and SETDB1 primarily modify H3K27, H3K36, and H3K9, while protein arginine methyltransferases (PRMTs), such as PRMT1 and PRMT5, influence transcriptional regulation and RNA splicing via arginine methylation [193,194].

One extensively studied HMT is EZH2, the catalytic component of the polycomb repressive complex 2 (PRC2), which mediates H3K27me3 and transcriptional silencing. Overexpression or mutation of *EZH2* has been associated with tumor progression and metastasis across several cancers [195,196]. In the context of bone metastasis, EZH2 promotes osteolytic lesion development by upregulating integrin β1 and activating FAK–TGF-β signaling, thus enhancing osteoclast differentiation and tumor growth within the skeletal niche [197].

Tazemetostat, an oral EZH2 inhibitor, is the first-in-class FDA-approved agent for epithelioid sarcoma and relapsed or refractory follicular lymphoma with EZH2 mutation [198]. It inhibits both mutant and wild-type EZH2, thereby reversing gene repression mediated by H3K27me3 and suppressing tumor proliferation [196]. However, in breast cancer metastasis models, particularly in bone, tazemetostat and similar EZH2 inhibitors such as GSK126 have shown limited efficacy, likely due to EZH2’s non-catalytic functions, which remain active despite inhibition of its methyltransferase activity [196]. This highlights the need for combinatorial strategies that target both enzymatic and non-enzymatic roles of EZH2 [128]. Notably, upstream regulators like YTHDF1, which enhance EZH2 translation, have emerged as potential therapeutic targets capable of reducing osteolytic bone metastases [128]. Conversely, in models of peritoneal metastasis in triple-negative breast cancer, EZH2-driven H3K27me3 activity was shown to paradoxically promote the transcription of pro-metastatic genes such as *KRT14*, underscoring the context-dependent and multifaceted roles of histone methyltransferases in tumor dissemination [196].

Histone demethylases (HDMs), which consist of the lysine-specific histone demethylase 1A (LSD1) also known as lysine-specific demethylase 1A (KDM1A) family, as well as the Jumonji-C (JmjC) domain-containing groups such as KDM4 and KDM6, are essential for the modulation of gene expression through the reversal of histone methylation modifications [194]. This process affects transcriptional dynamics linked to oncogenesis and metastasis [194]. These enzymes are critical regulators of various cancer-related activities, including cellular proliferation, programmed cell death (apoptosis), epithelial-mesenchymal transition (EMT), evasion of immune responses, and resistance to chemotherapy [163]. Specifically, members of the KDM4 family (KDM4AC) are responsible for the demethylation of H3K9me3 and H3K36me3, thereby facilitating chromatin restructuring, contributing to genomic instability, and enhancing hormone receptor signaling pathways [199].

In breast cancer, it has been observed that KDM4A, KDM4B, and KDM4C exhibit abnormal expression patterns that lead to increased rates of invasion, proliferation, and osteogenic differentiation, crucial characteristics for successful metastatic colonization, particularly in osseous tissues [199]. Furthermore, silencing KDM6A, an enzyme that targets H3K27me3, has been shown to inhibit metastatic migration, thereby emphasizing the nuanced, context-dependent roles of HDMs in regulating cellular plasticity and the organ-specific tendencies of metastasis [200].

Notably, the interplay between epigenetic enzymes and metabolic pathways adds further complexity [201]. A recent study by Luo et al. (2025) identified a *KDM1A*–*PIAS4*–*SLC7A11* axis that promotes ferroptosis resistance in breast cancer [201]. Inhibition of *KDM1A* by Tanshinone IIA (Tan IIA) or ORY-1001 suppressed this pathway, leading to increased ferroptosis and reduced tumor growth and lung metastases in vivo [201]. While not yet tested in bone metastasis models, these findings suggest that targeting *KDM1A* could also modulate redox homeostasis in bone-resident cancer cells, which are often resistant to oxidative stress.

Collectively, these studies underscore the complex and multifaceted roles of histone methylation dynamics in metastatic progression [192]. The ability of HMTs and HDMs to reprogram cancer cell epigenomes facilitates organotropic metastasis, with bone representing a particularly permissive niche, modulated by these enzymes [192]. The development of dual-function inhibitors or combination therapies that target both catalytic and non-catalytic epigenetic activities represents a promising strategy for future therapeutic interventions in metastatic cancer [198].

### 3.4. Bromodomain and Extra-Terminal Domain (BET) Inhibitors

Bromodomain and Extra-Terminal Domain (BET) proteins, which include BRD2, BRD3, BRD4, and BRDT, act as epigenetic readers that identify acetylated lysine residues on histone tails, through their conserved bromodomains (BD1 and BD2) [202]. This interaction facilitates the recruitment of transcriptional machinery and coactivators to chromatin, thereby enhancing the expression of oncogenic drivers, such as MYC and BCL2. BET inhibitors (BETi), including JQ1, I-BET, and BI 894999, competitively occupy the acetyl-lysine recognition sites within these bromodomains, resulting in the displacement of BET proteins from chromatin and the silencing of critical oncogenes [203]. The disruption of BRD4-P-TEFb complex formation hinders RNA polymerase II-mediated transcription elongation, particularly at genes associated with super-enhancers, which are often vital for tumor maintenance [202,203].

BET inhibitors have emerged as a promising category of epigenetic therapeutics that target transcriptional dependencies in cancer [204]. By binding to the bromodomains of BET proteins (BRD2, BRD3, BRD4, and BRDT), these inhibitors obstruct the recognition of acetylated histones, leading to the suppression of key oncogenes such as *MYC*, *BCL2*, and *TMPRSS2-ERG*, which are essential for tumor proliferation, survival, and resistance to therapy [205].

Clinically, BET inhibitors have demonstrated promising preclinical efficacy in various malignancies, including hematologic cancers, triple-negative breast cancer, and prostate cancer [202]. The lead clinical candidate, molibresib (GSK525762), showed partial responses in NUT midline carcinoma (NMC) a BRD4-fusion-driven malignancy in a phase I trial (NCT01587703), with manageable toxicity including thrombocytopenia and fatigue [206]. However, its limited efficacy in solid tumors such as castration-resistant prostate cancer (CRPC) and small cell lung cancer (SCLC) underscores the challenges posed by tumor heterogeneity and compensatory survival pathways [203,205,206].

Likewise, birabresib (OTX015), a selective BET inhibitor, was assessed in a phase Ib trial involving patients with relapsed/refractory non-Hodgkin lymphomas (NHLs), acute leukemias, and advanced solid tumors (NCT01713582) [207]. The trial indicated some durable responses in hematologic malignancies, particularly diffuse large B-cell lymphoma (DLBCL), although responses in solid tumors were rare [207,208]. The dose-limiting toxicities included thrombocytopenia and gastrointestinal side effects, which were generally reversible and manageable [207].

RO6870810 (CC-90010), another investigational BET inhibitor developed by Celgene/Bristol-Myers Squibb, has been evaluated in several phase I/II trials, both as a standalone treatment and in combination regimens [209]. In a phase I dose-escalation study (NCT03220347), RO6870810 was tested in solid tumors, DLBCL, and multiple myeloma [209,210]. While monotherapy demonstrated tolerable safety and some biological activity, significant clinical benefit was limited, leading to ongoing research into synergistic combinations, such as with immune checkpoint inhibitors or proteasome inhibitors [210].

Despite the absence of FDA-approved BET inhibitors, compounds like BI 894999 have progressed to phase Ia/Ib clinical trials involving patients with advanced solid tumors and hematologic malignancies, including diffuse large B-cell lymphoma (DLBCL) [203]. These trials have revealed a manageable safety profile, albeit with limited efficacy as standalone treatments, emphasizing the necessity for combination strategies [203].

Pelabresib (CPI-0610) has demonstrated the potential to modify disease progression in myelofibrosis (MF). In the Phase II MANIFEST trial, the combination of Pelabresib and ruxolitinib resulted in a ≥35% reduction in spleen volume for 68% of patients who were naïve to JAK inhibitors at the 24-week mark, alongside enhancements in symptoms, bone marrow fibrosis, and levels of inflammatory cytokines [211]. These findings bolster its continued assessment in the Phase III MANIFEST-2 trial, and underscore the therapeutic potential of BET inhibition in influencing fibrotic and inflammatory pathways associated with bone marrow disorders [211].

Although direct investigations into the effects of BET inhibitors on bone metastases are scarce, growing evidence indicates their potential in influencing metastatic progression through the transcriptional regulation of pro-metastatic genes [212]. For instance, *BRD4* has been identified as a promoter of epithelial–mesenchymal transition (EMT), invasion, and survival under conditions of metastatic stress by enhancing WNT signaling and interacting with transcription factors like TWIST and NF-κB. In models of castration-resistant prostate cancer (CRPC), the inhibition of BET proteins disrupted androgen receptor signaling and downregulated pro-metastatic genes such as *TMPRSS2-ERG*, highlighting their significance in the metastatic spread to bone sites [205]. Additionally, in metastatic ovarian cancer, the inhibition of BRPF1, a non-BET bromodomain protein, was found to diminish migration and invasion by modulating transcriptional networks associated with metastasis, including PPARα signaling [213].

Moreover, JQ1, a pioneering BET inhibitor that specifically targets BRD4, effectively disrupts the binding of acetyl-lysine, leading to the suppression of critical oncogenes such as *MYC*, *BCL2*, and *CCND1* [210]. This mechanism results in cell cycle arrest and apoptosis. In addition to its direct antitumor properties, JQ1 also plays a role in disrupting the tumor–bone osteolytic cycle [214]. Recent research has demonstrated that the co-delivery of JQ1 with the osteoprotective agent icaritin, utilizing a hypoxia-responsive, RGD-modified nanoparticle (ARNP), significantly inhibited breast cancer bone metastases, reduced osteolysis, and prevented secondary lung metastases [214]. This highlights JQ1’s dual ability to target both tumor growth and the bone metastatic environment [214].

### 3.5. Non-Coding RNA-Based Therapies

Non-coding RNAs (ncRNAs), which include microRNAs (miRNAs), long non-coding RNAs (lncRNAs), small interfering RNAs (siRNAs), and small nucleolar RNAs (snoRNAs), are increasingly recognized as crucial regulators in the processes of oncogenesis, metastasis, and resistance to therapy [215]. They are currently being investigated as both therapeutic agents and targets in cancer treatment [216].

#### 3.5.1. MicroRNA-Based Therapies

Therapeutic strategies involving microRNA (miRNA) mimics and antagomirs aim to correct the dysregulation of miRNA activity in cancer, particularly concerning the advancement and spread of bone metastases [115,217]. MiRNA mimics are synthetic double-stranded RNAs that aim to restore the function of downregulated tumor-suppressive miRNAs [217]. For example, miR-34a, a significant suppressor of metastatic characteristics such as epithelial–mesenchymal transition (EMT) and osteoclast activation, has demonstrated effectiveness in preclinical models of skeletal metastasis [218]. The reintroduction of miR-34a resulted in the inhibition of SIRT1 and VEGF signaling pathways, leading to a decrease in tumor burden and osteolysis [219,220]. Although MRX34, a liposomal miR-34a mimic, progressed to a phase I clinical trial (NCT01829971) for advanced solid tumors and hepatocellular carcinoma, the trial was discontinued due to immune-related toxicities, underscoring both the therapeutic promise and the delivery challenges associated with miRNA mimics [220].

In contrast, antagomirs, chemically modified antisense oligonucleotides, operate by suppressing oncogenic microRNAs (oncomiRs), which are excessively expressed in bone metastatic cancers [221]. Specifically, MRG-106 (Cobomarsen), an antagomir that targets miR-155, has progressed to early clinical trials for patients with cutaneous T-cell lymphoma, and may have broader implications for metastatic treatment [222,223].

Regarding bone metastasis, miR-21, miR-10b, and miR-19a have been identified as facilitators of osteoclastogenesis and metastatic colonization by interfering with tumor suppressor pathways and amplifying RANKL signaling [224,225]. Preclinical studies that inhibited these miRNAs using antagomirs resulted in reduced bone resorption and hindered the establishment of the pre-metastatic niche [212,225]. These results underscore the therapeutic potential of miRNA mimics and antagomirs in influencing critical pathways associated with bone metastasis, although further clinical application will necessitate the refinement of delivery systems and assessment of toxicity [225].

#### 3.5.2. LncRNAs, circRNAs and eRNAs as Therapeutic Targets

Long non-coding RNAs (lncRNAs) and circular RNAs (circRNAs) are increasingly acknowledged as vital regulators of gene expression and chromatin dynamics in cancer, particularly in the context of metastasis [226]. Novel therapeutic approaches are being developed to target these molecules using antisense oligonucleotides (ASOs), which specifically bind to lncRNA transcripts to promote RNase H-mediated degradation or to obstruct their interactions with chromatin and protein partners [227]. A prominent example is *HOTAIR*, a well-studied oncogenic lncRNA that recruits the PRC2 complex to epigenetically silence tumor suppressor genes [228,229]. Preclinical investigations have shown that the silencing of *HOTAIR* via ASOs diminishes invasion and metastasis in models of breast and prostate cancer [228,230]. Another strategy involves GapmeRs, which are single-stranded ASOs enhanced with locked nucleic acid (LNA) modifications to increase their stability and effectiveness [231]. GapmeRs that target MALAT1, a lncRNA linked to metastasis and unfavorable prognosis in lung and bone cancers, have demonstrated significant antitumor activity in preclinical studies, hindering cell migration and the formation of metastases [99,232].

In parallel, CircRNAs are gaining attention as innovative therapeutic targets, due to their distinctive covalently closed loop structure, which provides resistance to exonuclease degradation and enables them to function as sponges for oncogenic miRNAs [227]. By modulating critical signaling pathways such as Wnt/β-catenin, PI3K/AKT, and TGF-β, circRNAs play a significant role in processes like epithelial–mesenchymal transition (EMT), osteoclast activation, and metastatic colonization [233,234]. Their stable presence in exosomes and the bloodstream makes them promising non-invasive biomarkers for assessing skeletal metastatic risk and tracking treatment responses [235]. Additionally, recent developments in circRNA-based vaccine platforms have shown enhanced antigen presentation and immune activation in preclinical cancer models compared to traditional linear mRNA vaccines, indicating potential future applications in the context of metastatic disease [236].

In addition to lncRNAs and circRNAs, enhancer RNAs (eRNAs), a class of noncoding RNAs transcribed from enhancer regions, are emerging as important regulators of gene expression and cancer progression. Notably, the eRNA SLIT2 has been shown to inhibit bone metastasis in breast cancer by modulating the p38 MAPK/c-Fos signaling pathway, thereby suppressing tumor cell invasion and colonization within the bone microenvironment. These findings position eRNAs as promising therapeutic targets for the prevention and treatment of bone metastases [237].

#### 3.5.3. CRISPR/dCas9 Epigenome Editing

Simultaneously, sophisticated genome-editing technologies, including CRISPR-dCas9-based epigenome editing, are being developed to accurately regulate lncRNA expression without causing double-strand breaks [238]. These methodologies utilize catalytically inactive Cas9 linked to epigenetic effectors (such as KRAB or TET1) to either repress or activate lncRNA loci [239]. Initial proof-of-concept studies have effectively employed this approach to silence NEAT1 and PVT1, both of which play roles in metastatic signaling and therapeutic resistance [99,240]. Although therapies targeting lncRNAs are largely confined to preclinical research, no lncRNA- or circRNA-directed agents have yet received FDA approval, and no phase II or III clinical trials are currently registered.

#### 3.5.4. siRNAs and snoRNAs

Small interfering RNAs (siRNAs) are short, double-stranded RNA molecules that promote the sequence-specific degradation of target messenger RNAs (mRNAs), effectively silencing oncogenic drivers and genes associated with drug resistance [241]. Initially designed for the treatment of genetic disorders, siRNA-based therapies are now being adapted for use in oncology [215]. A notable example is siG12D-LODER, a biodegradable polymeric implant that delivers siRNA targeting the mutant KRASG12D, which has demonstrated encouraging outcomes in a phase I/II clinical trial (NCT01188785) for pancreatic ductal adenocarcinoma, resulting in tumor stabilization and enhanced survival rates [242].

Concurrently, small nucleolar RNAs (snoRNAs), which have been traditionally recognized for their role in guiding rRNA modifications, are now acknowledged as significant modulators of post-transcriptional gene regulation and epigenetic reprogramming in cancer [115]. Recent research has uncovered dysregulated snoRNAs, including SNORD50A/B and SNORD78, as facilitators of tumor progression and metastasis by influencing chromatin accessibility and interacting with oncogenic pathways [243,244]. In the context of bone metastases, Small nucleolar RNA host gene 3 (SNHG3), a long non-coding RNA associated with snoRNA clusters, has been shown to enhance bone metastasis in prostate cancer [245]. Higher SNHG3 expression is correlated with advanced disease and a worse prognosis [245]. It functions as a competitive endogenous RNA, sequestering miR-214-3p, which leads to TGFBR1 upregulation and activation of the TGF-β pathway, promoting metastasis [245]. These findings highlight SNHG3’s potential as a biomarker and therapeutic target in prostate cancer with bone metastasis [245].

### 3.6. Epigenetic Modulation of the Bone Microenvironment by Tumor-Derived Factors

Recent studies indicate that epigenetic modifications not only influence the inherent behavior of tumor cells, but also significantly alter the bone microenvironment, to facilitate metastatic colonization [246]. The process of bone remodeling is governed by the synchronized actions of osteoclasts and osteoblasts, with this intricate mechanism being epigenetically modulated through DNA methylation, histone modifications, and non-coding RNAs [159]. In the context of metastasis, particularly in breast and prostate cancers, signals derived from tumors, such as TGF-β, IL-6, and extracellular vesicles (EVs), trigger epigenetic changes in cells residing in the bone [159,246]. This results in the inhibition of osteoblastogenesis and the promotion of osteoclast differentiation via the modulation of the RANK/RANKL/OPG signaling pathway [247]. For example, cancer-associated fibroblasts (CAFs) present in the metastatic bone environment demonstrate DNA hypomethylation and modified histone acetylation, which enhance the release of pro-osteolytic factors such as MMPs and VEGF [248].

Furthermore, circular RNAs (circRNAs) and long non-coding RNAs (lncRNAs) have been recognized as regulators of osteoclastogenic gene expression by sequestering miRNAs or modifying chromatin accessibility, an area currently under extensive research [230,249]. These insights underscore how metastatic tumors leverage the plasticity of bone through epigenetically regulated mechanisms, thereby creating a conducive environment for skeletal colonization [249].

The reversibility of epigenetic modifications presents a compelling opportunity for therapeutic intervention [159]. Epigenetic drugs, including histone deacetylase inhibitors (HDACis), DNA methyltransferase inhibitors (DNMTis), and emerging bromodomain and extra terminal domain (BET) inhibitors, have demonstrated potential to normalize bone homeostasis and impair the metastatic cascade [173,191,250]. Recent studies suggest that these agents can be effectively combined with standard bone-targeted therapies such as bisphosphonates and denosumab, yielding synergistic effects in reducing skeletal-related events (SREs) and tumor burden [191].

Moreover, novel dual-function epigenetic agents capable of reactivating silenced tumor suppressors while simultaneously modulating osteoclastogenic signaling pathways are under preclinical development [159]. Innovative approaches also include the use of nanoparticle-based delivery systems to target epigenetic drugs specifically to the bone microenvironment, thereby minimizing off-target effects [210]. Importantly, patient-derived bone organoid models are now being employed to study bone-specific epigenetic signatures and drug responses in a physiologically relevant context [251]. As our understanding deepens, integrating epigenetic profiling of the bone metastatic niche with targeted therapeutics promises to advance precision oncology strategies aimed at disrupting the vicious cycle of bone metastasis [252].

### 3.7. Chromatin Remodeling and Its Role in Cancer Progression and Bone Metastasis

ATP-dependent chromatin remodeling complexes, particularly the mammalian SWI/SNF (mSWI/SNF or BAF) family, play a crucial role in regulating genome accessibility and transcriptional regulation [253]. These complexes remove nucleosomes by utilizing energy from ATP hydrolysis, thereby facilitating or hindering the access of transcription factors to DNA [253]. At the core of their functionality are the mutually exclusive ATPase subunits BRG1 (SMARCA4) and BRM (SMARCA2), which function as molecular motors that drive chromatin remodeling [254]. Although BRG1 and BRM exhibit significant sequence homology, their expression is influenced by context, and is specific to certain lineages [254]. BRG1 has been demonstrated to interact with TGF-β signaling pathways and to facilitate epithelial-to-mesenchymal transition (EMT), both of which are critical processes in metastatic progression, including metastasis to bone [253]. In the context of prostate cancer, BRG1 additionally regulates androgen receptor-mediated transcription and enhances looping mechanisms that are associated with bone metastasis [253,255]. The loss or mutation of BRG1/BRM disrupts normal enhancer–promoter interactions, which allows for the activation of oncogenic transcriptional programs and the acquisition of stem-like characteristics [255]. Importantly, mutations in mSWI/SNF subunits are found in over 20% of all human cancers, rendering these complexes among the most frequently altered chromatin regulators in the process of oncogenesis [255].

Recent findings have spurred the advancement of therapeutics targeting chromatin remodelers, with FHD-286, a selective dual BRG1/BRM ATPase inhibitor, emerging as a prominent candidate [256]. Preclinical investigations reveal that FHD-286 promotes chromatin compaction, inhibits oncogenic regulators such as c-Myc and PU.1, and diminishes leukemia-initiating potential in acute myeloid leukemia (AML) models [254]. This compound is presently undergoing clinical trials (NCT04891757), and its mechanism of action, focusing on BRG1/BRM-dependent transcription, positions it as a potential therapeutic for cancers characterized by chromatin remodeling vulnerabilities, including those susceptible to bone metastasis [254]. Furthermore, evidence from kidney and prostate cancer models indicates that alterations in SWI/SNF contribute to the osteotropic phenotype, through enhanced Wnt and TGF-β signaling [255]. Although specific trials for bone metastatic breast or prostate cancers are currently absent, the expanding array of SWI/SNF-targeting agents, including BRD9 degraders and SMARCA4/2 inhibitors, provides a basis for future translational research in this domain [257].

Epigenetic drugs work by modifying gene expression without changing the underlying DNA sequence. For example, BET inhibitors like JQ1 have been shown to effectively inhibit cancer cell growth and prevent osteosarcoma-related bone remodeling in preclinical models [250,258]. These inhibitors also target important signaling pathways that control osteoclastogenesis, emphasizing their role in maintaining bone balance while offering therapeutic effects against tumors in the bone microenvironment [259,260]. The interaction between bone-associated tumors and bone-resorbing cells highlights the complexities involved in treating such metastatic diseases.

A major challenge in targeting bone metastases with epigenetic therapies lies in the unique features of the bone microenvironment. Bone metastases often involve a cycle of tumor growth and bone resorption [250]. Tumor cells can alter the bone microenvironment, to enhance their survival and resistance to treatments [156]. This interaction necessitates the development of innovative therapies aimed at overcoming drug resistance, a significant obstacle in treating bone-related cancers. Recent research suggests that combining epigenetic drugs with traditional treatments may help reduce resistance, leading to more effective therapy options [261].

In the context of their therapeutic evaluation, several epigenetic drugs still require extensive preclinical testing, specifically targeting bone metastases. Studies have shown that BET inhibitors can influence inflammatory responses that contribute to bone resorption and metastasis [260,262]. For instance, the inhibition of BET proteins has been demonstrated to decrease levels of NF-κB target genes in macrophages, which are key players in the inflammatory environment of the bone microenvironment [262]. Moreover, combining BET inhibitors with other therapeutic agents has resulted in enhanced effectiveness against resistant cancer cells present in bone metastases [263].

Furthermore, the physical features of the bone environment can affect drug delivery processes. Bisphosphonates (BPs) are used in the management of bone metastasis because they can selectively bind to bone and alter the tumor microenvironment [264]. Research shows that using BP-conjugated chemotherapeutics can effectively target therapy to the site of action, potentially overcoming some pharmacological obstacles created by the bone microenvironment in metastatic disease [264].

It is crucial to recognize that issues related to drug bioavailability and resistance are not limited to any specific type of epigenetic agent. Developing acquired resistance to DNMT and HDAC inhibitors, for example, remains a significant challenge that hampers the effectiveness of these promising drugs [263]. Understanding genetic and epigenetic changes in metastatic tumors is essential for refining therapy strategies. Evidence suggests that specific epigenetic modifications can render cells more resistant to treatment, underscoring the need for combination strategies to overcome this resistance. Specifically, the genetic makeup of bone metastases often requires personalized medicine approaches that incorporate genomic profiling to inform treatment decisions [156,265].

Recent explorations into nanomedicine have also revealed innovative strategies to enhance therapeutic effectiveness against bone tumors. Engineered nanoparticles designed for bone targeting can deliver epigenetic drugs locally, potentially overcoming some issues related to systemic side effects and strengthening localized drug effects [214,261]. These nanoparticles can significantly alter the behavior of epigenetic drugs in the body, opening up new avenues to enhance their effectiveness for treating bone metastases.

While evaluating epigenetic drugs like DNMT, HDAC, and BET inhibitors in preclinical models of bone metastases shows promising therapeutic potential, significant challenges remain with drug resistance and delivery methods. Ongoing research is essential to determine the best ways to use these drugs for bone tumor treatment, effectively. By targeting the specific features of the bone microenvironment, researchers can develop better strategies to address the limitations of traditional therapies.

Table 1 provides a detailed summary of clinically significant epigenetic modulators, which include DNMT inhibitors, HDAC inhibitors, histone methyltransferase and demethylase inhibitors, BET inhibitors, and RNA-based therapies. These agents have emerged as promising tools in combating cancer progression and bone metastasis [252]. In addition to reactivating silenced tumor suppressor genes, they influence the tumor microenvironment and interfere with critical metastatic signaling pathways within the bone niche [159].

Despite encouraging outcomes in preclinical and early-phase clinical studies, several challenges remain, such as off-target toxicity, resistance, and limited efficacy in solid tumors [14,252]. These limitations underscore the need for optimized combinatorial strategies and advanced delivery systems tailored to bone metastases [159,212].

In summary, while agents like tazemetostat (EZH2 inhibitor), azacitidine, decitabine, and various HDAC inhibitors are already FDA-approved for hematologic or related malignancies, their potential application in bone-predominant metastatic settings is actively being explored [173,184,198,266]. Investigational compounds such as entinostat, pelabresib, and nanoparticle-formulated JQ1 have shown promise by targeting the bone microenvironment and suppressing metastatic outgrowth signaling [185,211,214]. Addressing the remaining challenges will be essential for translating these therapies into clinical success in metastatic bone disease.

**Table 1:** Summary of clinically relevant epigenetic modulators.

Drug	Class	Cancer Type/Indication	Status	Clinical Trial Phase/Approval	Reference
5-Azacytidine	DNMT inhibitor	MDS, AML, brain metastasis	FDA-approved	Approved	[166,175]
Decitabine	DNMT inhibitor	MDS, AML, bone metastasis	FDA-approved	Approved	[173]
Guadecitabine (SGI-110)	SGI—Second-generation DNMT inhibitor	Advanced solid tumors	In trials	Phase I	[176]
GSK3685032	DNMT selective inhibitor	AML	Preclinical	Preclinical	[177]
Vorinostat	HDAC inhibitor	Cutaneous T-cell lymphoma	FDA-approved	Approved	[267]
Romidepsin	HDAC inhibitor	Peripheral T-cell lymphoma	FDA-approved	Approved	[183]
Panobinostat	HDAC inhibitor	Multiple myeloma	FDA-approved	Approved	[268]
Belinostat	HDAC inhibitor	Peripheral T-cell lymphoma	FDA-Approved	Approved	[184]
Entinostat	HDAC inhibitor	Breast cancer	In trials	Breakthrough therapy	[185]
Mocetinostat	HDAC inhibitor	Solid tumors	In trials	Phase I/II	[182]
Givinostat	HDAC inhibitor	Polycythemia vera, leukemia	In trials	Phase III	[187]
Abexinostat	HDAC inhibitor	Lymphoma, solid tumors	In trials	Phase II	[188]
Tubastatin A	HDAC6 inhibitor	Preclinical cancers	Preclinical	Preclinical	[84]
PCI-34051	HDAC8 inhibitor	Specific cancers	Preclinical	Preclinical	[190]
REC-2282 (AR-42)	HDAC inhibitor	Neurofibromatosis type 2, meningioma	In trials	Phase I/II	[186]
Tazemetostat	EZH2 inhibitor	Follicular lymphoma, epithelioid sarcoma	FDA-approved	Approved	[198]
GSK126	EZH2 inhibitor	Solid tumors	Preclinical	Preclinical	[196]
ORY-1001	KDM1A inhibitor	AML, breast cancer	Preclinical	Preclinical	[201]
Tan IIA	KDM1A natural inhibitor	Breast cancer	Preclinical	Preclinical	[201]
JQ1	BET inhibitor	Breast cancer bone metastasis	Preclinical	Preclinical	[210]
Molibresib	BET inhibitor	NUT midline carcinoma	In trials	Phase I	[206]
Birabresib (OTX015)	BET inhibitor	NHL, AML	In trials	Phase Ib	[207]
RO6870810	BET inhibitor	DLBCL, solid tumors	In trials	Phase I/II	[209]
BI 894999	BET inhibitor	DLBCL, solid tumors	In trials	Phase Ia/Ib	[203]
Pelabresib (CPI0610)	BET inhibitor	Myelofibrosis	In trials	Phase II/III	[211]
MRX34	miR-34a mimic (RNA-based therapy)	Liver cancer, melanoma	Terminated	Phase I (terminated)	[220]
MRG-106 (Cobomarsen)	Antagomir (miR-155)	Cutaneous T-cell lymphoma	In trials	Phase I	[222,223]
siG12D- LODER	siRNA	Pancreatic cancer	In trials	Phase I/II	[242]
FHD-286	BRG1/BRM dual ATPase inhibitor	AML, SWI/SNF-mutant cancers	In trials	Phase I	[254]

## 4. Future Direction

Advancing the epigenetic understanding of bone metastasis demands a convergence of cutting-edge technologies and biologically faithful models. Single-cell and spatial epi-omics—encompassing high-resolution methylome and chromatin architecture profiling—promise to uncover micro-anatomical niches of epigenetic vulnerability within tumor, stromal, and immune compartments, which are often obscured in bulk tissue analyses. When integrated with spatial transcriptomics, these atlases could illuminate lineage-specific epigenetic dependencies suitable for therapeutic targeting. In parallel, the development of patient-derived bone organoids and microfluidic “osteon-on-a-chip” platforms that recapitulate osteoblast, osteoclast, and marrow stromal crosstalk under physiologic shear forces and mineral gradients will offer a powerful ex vivo system for screening epigenetic drug combinations. To enhance therapeutic precision, bone-targeted delivery systems—such as hydroxyapatite-binding peptides and bisphosphonate-linked nanoparticles—are being explored, to selectively concentrate DNMT, BET, or KDM inhibitors within skeletal lesions, thereby minimizing systemic toxicity. Moreover, combinatorial regimens that integrate epigenetic agents with immune checkpoint blockades (e.g., PD-1/PD-L1, CTLA-4) are gaining momentum, leveraging the capacity of epigenetic priming to restore antigen presentation and viral mimicry pathways. Future clinical trials should prioritize bone-specific endpoints, such as time to first skeletal-related event. An emerging frontier also lies in the metabolic–epigenetic interface, where tumor-derived metabolites—including lactate, glutamine, and oncometabolites like α-ketoglutarate—modulate the activity of chromatin-modifying enzymes. Therapeutic strategies that couple metabolic inhibitors (e.g., IDH or glutaminase blockers) with epigenetic drugs may yield synergistic suppression of metastatic progression. Finally, locus-specific epigenome editing via CRISPR/dCas9 fused to chromatin-modifying domains enables scar-free activation of metastasis suppressors or silencing of osteolytic drivers, with early proof-of-concept studies targeting NEAT1 and PVT1. However, ensuring off-target safety and achieving bone-selective delivery remain essential hurdles for clinical translation.

## 5. Conclusions

Epigenetic plasticity endows disseminated tumor cells with the dynamic capacity to adapt to, and ultimately subvert, the bone microenvironment. Aberrations in DNA methylation patterns, histone modifications, chromatin remodeling complexes, and non-coding RNA networks not only enhance the invasive and stem-like properties of cancer cells, but also reprogram osteoblasts, osteoclasts, and marrow stromal cells to establish and sustain a pro-metastatic niche. From a therapeutic standpoint, first-generation epigenetic drugs—such as hypomethylating agents and histone deacetylase inhibitors—have demonstrated the tractability of chromatin as a drug target. However, their clinical impact is often constrained by off-target toxicities, limited specificity, and the ability of tumor cells to exploit microenvironmental escape routes.
